# Clinical Impact and Cost-effectiveness of Xpert MTB/RIF Testing in Hospitalized Patients With Presumptive Pulmonary Tuberculosis in the United States

**DOI:** 10.1093/cid/ciw803

**Published:** 2016-12-10

**Authors:** James F. Cowan, Aldine S. Chandler, Elizabeth Kracen, David R. Park, Carolyn K. Wallis, Emelline Liu, Chao Song, David H. Persing, Ferric C. Fang

**Affiliations:** 1 Department of Global Health, University of Washington Schools of Medicine and Public Health; 2 Department of Medicine, University of Washington School of Medicine; 3 Harborview Medical Center; 4 Department of Laboratory Medicine, University of Washington School of Medicine, Seattle; 5 Cepheid, Sunnyvale, California; 6 Department of Microbiology, University of Washington School of Medicine, Seattle

**Keywords:** tuberculosis, airborne infection isolation, GeneXpert MTB/RIF, cost-effectiveness analysis

## Abstract

**Background.:**

Microscopic examination of acid-fast-stained sputum smears is the current standard of care in the United States to determine airborne infection isolation (AII) of inpatients with presumptive pulmonary tuberculosis (PTB). However, nucleic acid amplification testing (NAAT) with the Xpert MTB/RIF assay (Xpert) may be more efficient and less costly.

**Methods.:**

This prospective observational cohort study enrolled a consecutive sample of 318 AII-eligible inpatients from a public hospital in Seattle, Washington, from March 2012 to October 2013. Sputum samples were collected from each inpatient and analyzed using smear microscopy, culture, drug susceptibility testing, and NAAT. The performance, clinical utility (AII duration and survival), and cost-effectiveness from an institutional perspective were compared for 5 testing strategies.

**Results.:**

Among the 318 admissions with presumptive PTB, 20 (6.3%) were culture-positive for *Mycobacterium tuberculosis*. The sensitivity of 1 Xpert, 2 Xperts, 2 smears, or 3 smears compared to culture was 0.85 (95% confidence interval [CI], .61–.96), 0.95 (95% CI, .73–1.0), 0.70 (95% CI, .46–.88), and 0.80 (95% CI, .56–.93), respectively. A cost-effectiveness analysis of the study results demonstrated that an Xpert test on 1 unconcentrated sputum sample (assuming equivalent results for unconcentrated and concentrated sputum samples) is the most cost-effective strategy (99.9% preferred at willingness-to-pay of US$50000) and on average would save 51.5 patient-hours in AII and up to $11466 relative to microscopy without a compromise in sensitivity.

**Conclusions.:**

In hospitalized patients with presumptive PTB in a low-burden setting, NAAT can reduce AII and is comparably sensitive, more specific, and more cost-effective than smear microscopy.

## Summary

In hospitalized patients with presumptive pulmonary tuberculosis in a low-burden setting such as the United States, GeneXpert MTB/RIF molecular testing can reduce the duration of airborne infection isolation and is comparably sensitive, more specific, and more cost-effective than smear microscopy.

Tuberculosis remains a global problem, including in the United States. In 1989, the Centers for Disease Control and Prevention (CDC) set a goal of eliminating tuberculosis in the United States, defined as <1 case per million individuals [1]. However, in 2014, the CDC reported 9421 new US cases (3.0 cases per 100000 individuals), substantially in excess of the target [2].

For inpatients with presumptive pulmonary tuberculosis (PTB), CDC guidelines currently recommend collection of 3 respiratory specimens at 8- to 24-hour intervals, including at least 1 early-morning specimen, with testing by smear microscopy for acid-fast bacilli (AFB) and culture for *Mycobacterium tuberculosis* (MTB) [3]. Although this approach has reduced tuberculosis transmission in healthcare settings by directing the need for airborne infection isolation (AII), the smear-based strategy suffers from low sensitivity, a requirement for specialized training, and delayed results [4]. In 2009, recognizing the limitations of smear-based diagnosis, the CDC recommended that nucleic acid amplification testing (NAAT) be performed on at least 1 sputum specimen from patients with presumptive PTB, regardless of smear results. Despite this recommendation, NAAT-based testing is inconsistently performed in the United States due to a lack of supporting clinical and economic evidence [5–8].

The Xpert MTB/RIF assay (Cepheid, Sunnyvale, California) is a fully automated NAAT that can deliver a result for MTB and rifampin resistance in about 2 hours. In contrast to other NAATs, Xpert can be performed on-demand by personnel with minimal training [9, 10]. On the basis of studies in countries with high tuberculosis burdens, Xpert has been endorsed by the World Health Organization (WHO) and widely deployed [10–16]. However, few practical studies have assessed whether Xpert is cost-effective in low-burden, high-income countries [5, 17–20]. Several studies have measured the time required to collect and analyze sputum samples in an inpatient setting and documented low sensitivity and specificity of smear microscopy [21–24]. A recent systematic review has also shown that patients in respiratory isolation experience more adverse events, a negative impact on mental health, lower satisfaction, and reduced provider contact [25].

Xpert has been recently cleared for tuberculosis diagnosis by the US Food and Drug Administration (FDA) [26, 27]. We hypothesized that Xpert might reduce the costs of evaluating patients with presumptive PTB in a low-burden setting by reducing the need for AII.

## METHODS

### Study Setting

Harborview Medical Center (HMC) is a 413-bed public hospital in Seattle, Washington. Each year approximately 200 inpatients are placed in AII for presumptive PTB. Of these, <10% are ultimately culture-positive for MTB. HMC infection control policies require patients with presumptive PTB to remain in AII until 3 consecutive negative AFB smears 8–24 hours apart are obtained and analyzed.

### Study Population

**Figure 1.**
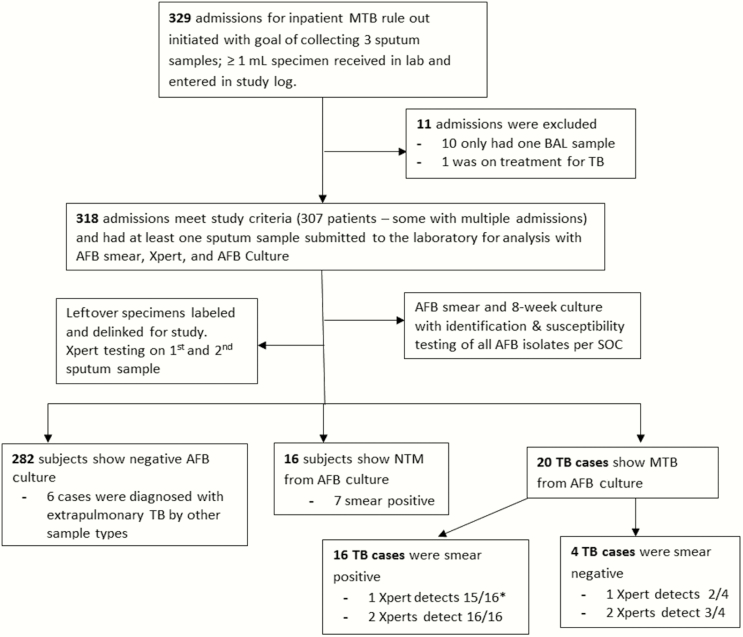
Study design, flowchart of inclusion and exclusion of study subjects, and summary of results. *One TB case was classified as 1+ smear positive on 1 of 3 sputum samples, and this case was reported as Xpert MTB negative on the first sputum sample but Xpert MTB-positive on the second sputum sample. Abbreviations: AFB, acid-fast bacilli; BAL, bronchoalveolar lavage; MTB, *Mycobacterium tuberculosis*; NTM, *nontuberculous mycobacteria*; SOC, standard of care; TB, tuberculosis; Xpert, Xpert MTB/RIF assay.

A consecutive cohort of patients admitted to HMC between March 2012 and October 2013 was enrolled. Each patient underwent evaluation for PTB at admission with submission of at least 1 sputum sample (≥1 mL) for AFB smear, and culture including Mycobacteria Growth Indicator Tube up to 6 weeks, Middlebrook 7H11 agar up to 8 weeks, and additional methods for speciation and drug susceptibility. Patients receiving ongoing treatment for tuberculosis were excluded. Laboratory records and medical charts were reviewed with approval from an institutional review board. Study design and a patient flowchart are shown in Figure 1.

### Laboratory Methods

For each patient, 1–3 sputum samples were collected at 8- to 24-hour intervals. Testing was performed in the HMC Clinical Microbiology Laboratory. Each sample (≥1 mL) was decontaminated and concentrated for AFB smear (auramine O) and culture. A leftover aliquot (0.5 mL) reserved for Xpert testing was bar coded and de-identified but linked to the patient record through a study identification number. Xpert was performed on the first and the second samples. Medical laboratory scientists were blinded to the results of smear and culture. Detailed procedures are provided in the Supplementary Materials.

### Diagnostic Strategies and Airborne Infection Isolation

Five strategies to guide AII were compared: (1) 1 Xpert on an unconcentrated sputum sample; (2) 1 Xpert on a concentrated sample; (3) 2 Xperts on concentrated sputum samples; (4) 2 smears; and (5) 3 smears. For the study it was assumed that AII could be discontinued on the basis of negative results for 1 Xpert (unconcentrated or concentrated), 2 consecutive Xperts, 2 consecutive smears, or 3 consecutive smears. Because Xpert was approved only for research use at the time the study was initiated, only smear and culture results were reported to clinicians. The 3-smear strategy was used as the actual basis for clinical decisions to discontinue AII (recorded electronically in the patient’s chart). For other strategies, hypothetical AII duration was estimated based on assumptions in Supplementary Table 1.

For this study, all samples were tested by Xpert after they were concentrated, which is required for all currently approved smear protocols to determine the need for AII in the United States. The cost analysis assumed that test performance results would be the same for unconcentrated and concentration sputum samples, which has subsequently been validated [10, 28].

### Analysis of Test Results and Duration of Airborne Infection Isolation

The performance of testing strategies was compared with that of culture (the gold standard for PTB diagnosis) or 3 sputum smears (the gold standard for AII discontinuation). A survival analysis compared AII duration for the 5 testing strategies. Kaplan-Meier survival curves and the medians with interquartile range of AII duration were determined. Kolmogorov-Smirnov test and log-rank were used to detect significant differences between survival curves. The times from AII initiation to submission of the first, second, and third samples for testing were also analyzed by survival analysis to estimate the lower bound of AII duration for various testing strategies.

### Cost-effectiveness Analysis

A CEA was conducted using TreeAge Pro Edition 2015. All enrolled subjects’ data were included. The cost-effectiveness analysis (CEA) was performed from the institutional perspective, and a decision tree analytical model was developed (summarized in Supplementary Figure 1), with the time horizon spanning from the time PTB was first suspected and AII initiated to the time of discharge. Clinical and cost inputs of the model and their ranges and sources are summarized in Supplementary Table 2. Where possible, inputs are observations from this study, and other inputs are based on assumptions from previous studies. Effectiveness was measured as correctly diagnosed positive cases, correctly diagnosed negative cases, and diagnostic accuracy. The primary outcome of the CEA was the incremental cost-effectiveness ratio (ICER), calculated as the differential cost of the base strategies divided by the differential diagnostic accuracy.

One- and 2-way probabilistic sensitivity analyses were performed to identify the most influential model parameters and to test the robustness of the model. Further details of the methods and results of this CEA analysis and the sensitivity analysis are included in the Supplementary Materials.

## RESULTS

### Patient Population

From March 2012 to October 2013, 329 patients were admitted to HMC for presumptive PTB. Eleven were excluded after chart review: 10 had only a single bronchoalveolar lavage sample submitted for analysis, and 1 patient was on a 4-drug therapeutic regimen for disseminated tuberculosis at the time of admission. Three hundred eighteen admission events were enrolled in this study (307 individual patients: 9 patients were admitted twice, and 1 was admitted 3 times during the course of the study).

Table 1 summarizes patient characteristics for the 318 admissions. Mean patient age was 50.3 years, and 78.3% were male; 129 (41.0%) were foreign-born with 123 from a high-risk region (Asia, Africa, Central and South America); 97 (30.7%) were homeless; 76 (23.9%) were HIV-infected; 66 (20.8%) were diabetic. During the study, 289 (91%) of patients had 3 sputum specimens collected, 16 (5%) had 2 specimens collected, and 13 (4%) had 1 specimen collected. For testing strategies that involved analyzing results from 1 sample, we included data from all 318 admission events. For testing strategies that involved analyzing results from 2 samples, we included data from the 305 admission events for which 2 or more sputum samples were collected. Patients had <3 sputum specimens collected for a variety of reasons, including (1) death; (2) early discharge; and (3) inability to get additional adequate sputum samples. No admission events were censored, as there were clear AII end points for each admission, including 3 patients that were never put in AII due to low clinical suspicion for tuberculosis and whose AII duration was recorded as zero.

**Table 1.** Patient Characteristics (N = 318)

Characteristic	All (N = 318)		PTB Cases (n = 20)		Non-PTB Cases (n = 298)		NTM Cases (n = 16)	
Days of isolation time, mean (SD)	3.14	(3.19)	7.06	(7.69)	2.88	(2.44)	5.69	(7.19)
Days of hospital stay, mean (SD)	8.44	(11.27)	7.75	(8.81)	8.48	(11.43)	14.44	(15.41)
Only 1 sputum specimen collected	13	(4.09)	0	(0)	13	(4.36)	0	(0)
Only 2 sputum samples collected	16	(5.03)	4	(20)	12	(4.03)	2	(12.5)
All 3 sputum specimens collected	289	(90.88)	16	(80)	273	(91.61)	14	(87.5)
No. of female cases	69	(21.7)	5	(25)	235	(78.86)	2	(12.5)
No. of male cases	249	(78.3)	15	(75)	63	(21.14)	14	(87.5)
Average age (range)	50	(18–88)	47	(26–86)	51	(18–88)	61	(31–82)
Fever	153	(48.11)	11	(55.00)	142	(47.65)	5	(31.25)
Cough	234	(73.58)	17	(85.00)	217	(72.82)	11	(25.00)
Night sweats	114	(35.85)	11	(55.00)	103	(34.56)	7	(43.75)
Weight loss	118	(37.11)	17	(85.00)	101	(33.89)	8	(50.00)
HIV positive	76	(23.9)	1	(5)	75	(25.17)	3	(18.75)
Homeless^a^	97	(30.7)	3	(15.79)	1	(0.34)	2	(12.5)
Born in a high-risk country	123	(38.68)	18	(90)	105	(35.23)	7	(43.75)
Foreign born	129	(40.57)	18	(90)	111	(37.25)	7	(43.75)
Diabetes	66	(20.75)	10	(50)	56	(18.79)	5	(31.25)
Admitted only for PTB rule-out	33	(10.38)	5	(25)	28	(9.4)	2	(12.5)

Data are presented as No. (%) unless otherwise indicated.

Abbreviations: HIV, human immunodeficiency virus; NTM, nontuberculous mycobacteria; PTB, pulmonary tuberculosis; SD, standard deviation.

a Two missing information regarding homelessness.

A total of 20 (6.3%) patients were diagnosed with active PTB on the basis of a positive MTB culture. Patients with confirmed active PTB were more likely to report weight loss, diabetes, or birth in a high-risk country, and less likely to be HIV-infected or homeless (Table 1). The mean AII duration was 3.1 days for all admissions, 2.9 days for PTB-negative cases, 7.1 days for confirmed PTB, and 5.7 days for nontuberculous mycobacterial (NTM) infections. The average admission duration was 8.4 days and was similar for PTB and non-PTB cases but substantially longer for NTM cases (14.4 days).

### Test Performance

The sensitivity, specificity, positive predictive value (PPV), and negative predictive value (NPV) of each testing strategy relative to culture are presented in Table 2. The highest sensitivity was observed for 2 Xperts, followed by 1 Xpert, 3 smears, and 2 smears, respectively. The specificities of the 5 strategies were similar (0.97–1.00), with slightly higher specificity observed for Xpert compared with smear. The NPV of the 5 strategies was also similar (0.99 for 3 smears, 0.98 for 2 smears, 0.99 for 1 Xpert, 1.00 for 2 Xperts). However, the PPV was substantially superior for Xpert-based strategies: 1.00 for 1 Xpert and 0.95 for 2 Xperts; compared with 0.64 for 3 smears and 0.70 for 2 smears, due to several patients with positive AFB smears from NTM infections.

**Table 2.** Performance of Airborne Infection Isolation Testing Strategies: 3 Acid-Fast Bacilli (AFB) Smears, 2 AFB Smears, 1 Xpert and 2 Xperts for Presumptive Tuberculosis Compared to *Mycobacterium tuberculosis* Culture

Testing Strategy	Sensitivity	(95% CI)	Specificity	(95% CI)	PPV	(95% CI)	NPV	(95% CI)
Overall (20 MTB culture-positive cases)								
3 smears	0.80	(.56–.93)	0.97	(.94–.99)	0.64	(.43–.81)	0.99	(.96–1.00)
2 smears	0.70	(.46–.88)	0.98	(.96–.99)	0.70	(.46–.90)	0.98	(.96–.99)
1 Xpert^a^	0.85	(.61–.96)	1.00	(.98–1.00)	1.00	(.77–1.00)	0.99	(.97–1.00)
2 Xperts	0.95	(.73–1.00)	1.00	(.98–1.00)	0.95	(.73–1.00)	1.00	(.98–1.00)
In 3 AFB smear positive (16 MTB culture-positive cases)								
2 smears	0.88	(.62–.98)	0.33	(.07–.70)	0.70	(.46–.88)	0.60	(.15–.95)
1 Xpert^a^	0.94	(.70–1.00)	1.00	(.63–1.00)	1.00	(.78–1.00)	0.90	(.56–1.00)
2 Xperts	1.00	(.80–1.00)	1.00	(.66–1.00)	1.00	(.80–1.00)	1.00	(.66–1.00)
In 3 AFB smear negative (4 MTB culture-positive cases)								
2 smears	…	…	1.00	(.99–1.00)	…	…	0.99	(.96–1.00)
1 Xpert^a^	0.50	(.07–.93)	1.00	(.99–1.00)	1.00	(.16–1.00)	0.99	(.98–1.00)
2 Xperts	0.75	(.19–.99)	1.00	(.98–.99)	0.75	(.19–.99)	1.00	(.98–.99)

Confidence intervals were calculated using the exact (Clopper-Pearson) confidence limits for the binomial proportion.

Abbreviations: CI, confidence interval; MTB, *Mycobacterium tuberculosis*; NPV, negative predictive value; PPV, positive predictive value; Smears, sputum smear microscopy; Xpert, Xpert MTB/RIF.

a Test performance of 1 Xpert unconcentrated and 1 Xpert concentrated is assumed to be the same in the cost-effectiveness analysis based on published studies [10, 28]. The performance reported here is from 1 Xpert concentrated.

Twenty-four patients had at least 1 positive AFB smear result. Of these, 16 were confirmed to have MTB by culture, and 8 harbored NTM. Four patients with negative smears grew MTB from culture. No MTB isolates were found to be resistant to first-line tuberculosis drugs by standard drug susceptibility testing. A single Xpert identified 17 of 20 culture-confirmed MTB cases (15 AFB sputum smear-positive cases and 2 smear-negative cases). Xpert identified 1 patient with PTB as having rifampin resistance, but this was subsequently determined to be a false-positive result based on phenotypic susceptibility testing of the MTB isolate and sequence analysis of the *rpoB* gene. The false-positive rifampin result was attributed to the presence of a synonymous *rpoB* mutation. Two Xperts identified an additional smear-positive culture-confirmed case (with 1 of 3 samples 1+ AFB smear-positive), and 1 additional smear-negative MTB case.

### Duration of Airborne Infection Isolation

**Figure 2.**
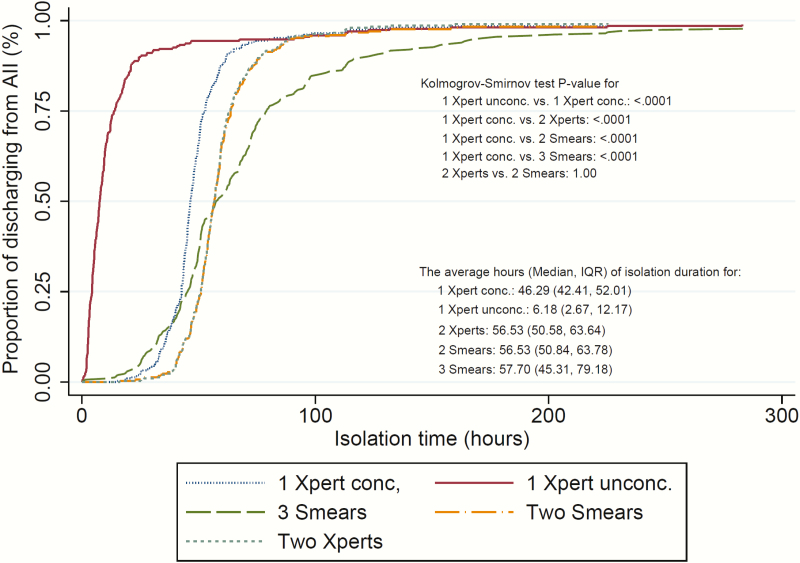
Kaplan–Meier curves of AII duration by 5 testing strategies: 3 smears, 2 smears, 1 Xpert concentrated, 1 Xpert unconcentrated, and 2 Xperts. Abbreviations: AII, airborne infection isolation; conc., concentrated sputum sample; IQR, interquartile range; Smears, acid-fast bacilli sputum smear microscopy; unconc, unconcentrated; Xpert, Xpert MTB/RIF assay.

After clinical evaluation for PTB at admission, patients were placed in AII. The first sputum specimens were submitted to the laboratory a median of 4.1 (IQR, 0.7–9.1) hours later. The second samples were submitted a median of 14.3 (IQR, 9.3–19.7) hours from AII initiation, and the third samples were submitted a median of 23.9 (IQR, 18.0–33.1) hours from AII initiation (Supplementary Figure 3). A typical patient without PTB remained in AII for 2.9 days awaiting 3 negative smears (Table 1). The survival analysis of AII duration showed that a strategy based on a single Xpert on either unconcentrated or concentrated sputum could significantly reduce AII duration in comparison to 2 Xperts, 2 smears, or 3 smears on concentrated samples (*P* < .001 for both Kolmogorov-Smirnov test and log-rank test). Specifically, the median duration of AII would be 6.2 (IQR, 2.7–12.2) hours for 1 Xpert on an unconcentrated sputum sample, 46.3 (IQR, 42.4–52.0) hours for 1 Xpert on a concentrated sample, 56.5 (IQR, 50.6–63.6) hours for 2 Xperts on concentrated samples, 56.5 (IQR, 50.8–63.8) hours for 2 smears, and 57.7 (IQR, 45.3–79.2) hours for 3 smears (Figure 2). AII differences for 2 Xpert, 3 Xpert, or smear with concentrated samples are modest, as most of these samples were batched, analyzed, and reported at the same time.

### Cost-effectiveness

The base case analysis showed that the 1 Xpert unconcentrated strategy would save $7002, $8889, $ 9082, or $11446 compared with 1 Xpert concentrated, 2 Xperts, 2 smears, and 3 smears per PTB suspect, respectively. One Xpert also had a higher probability of detecting true PTB cases and excluding non-PTB cases. As a result, 1 Xpert unconcentrated was cost-saving at an ICER of –$320893 compared with 2 smears and –$363987 compared to 3 smears. Compared to 2 Xperts, the 1 Xpert concentrated strategy would save $1887 per case of suspected PTB, with a slightly lower probability of detecting PTB cases but a higher probability of excluding non-PTB cases. The 2-Xpert strategy is more expensive but still cost-effective, with an ICER of $73926 compared with 3 smears (results shown in Table 3).

**Table 3.** Cost-effectiveness Analysis of Base Case of 5 Testing Strategies to Determine Need for Airborne Infection Isolation

Outcome Measure	Strategy				
	1 Xpert Unconcentrated^a^	1 Xpert Concentrated	2 Xperts Concentrated	2 Smears	3 Smears
Cost, US$					
Laboratory cost	116.00	116.00	231.99	13.59	20.38
Penalty for false negative	11.44	11.44	3.81	22.89	15.26
AII cost^b^	3444.31	10447.02	12225.62	12612.08	14967.03
Non-AII hospitalization cost^c^	16290.79	16290.79	16290.06	16295.88	16306.01
Total cost	19862.55	26865.26	28751.49	28944.44	31308.68
Incremental cost	…	7002.71	8888.94	9082.44	11446.13
Effectiveness					
% PTB case detected	0.053	0.053	0.060	0.044	0.05
% Non-PTB cases excluded	0.937	0.937	0.934	0.918	0.909
% Accuracy of diagnoses	0.991	0.991	0.994	0.962	0.959
Incremental effectiveness, accuracy diagnoses	…	0	0.003	−0.028	−0.031
	…	…	…	−0.031^d^	−0.035^d^
ICER, US$/accurate diagnosed case	…	…	2826682	−320893.45	−363986.93
	…	…	…	−6136^d^	−73926^d^

Abbreviations: AII, airborne infection isolation; ICER, incremental cost-effectiveness ratio; PTB, pulmonary tuberculosis; Smears, sputum smear microscopy; Xpert, Xpert MTB/RIF.

a Test performance of 1 Xpert unconcentrated and 1 Xpert concentrated is assumed to be the same in this cost-effectiveness analysis.

b AII cost includes cost for AII and treatment cost during AII.

c Non-AII hospitalization cost includes cost for AII, other diagnostics and treatment during non-AII.

d Compared with 2 Xpert concentrated strategy.

One-way sensitivity analyses (Supplementary Figures 4 and 5) revealed that the primary determinant of cost savings with the 1 Xpert strategies was a reduction in AII duration, which also impacted the overall duration of hospital stay. The superior sensitivity and specificity of Xpert, together with the low prevalence of PTB, was also more influential than other variables in determining the cost savings for a 1-Xpert strategy compared with smear. Notably, Xpert obviated the need for AII in patients with NTM, whereas smears did not. This is of particular importance in a low-burden setting, as the prevalence of NTM is higher than that of PTB in the United States [29]. Monte Carlo probabilistic sensitivity analyses demonstrated that a 1 Xpert unconcentrated strategy is more cost-effective than the other 4 strategies (99.99% of the time at a willingness-to-pay of US$50000) (Supplementary Figure 2).

## DISCUSSION

In this single-center observational cohort study and CEA, Xpert-based strategies decreased AII and cost compared with the standard of care, 3 AFB smears, in a high-resource, low-burden inpatient setting. Three smears detected only 80% of confirmed PTB cases, with a median AII time of 57.7 hours. Two Xperts resulted in a median AII time of 56.5 hours while detecting 100% of smear-positive cases and 75% of smear-negative, culture-positive cases. A 1 Xpert concentrated sputum strategy reduced median AII time to 46.3 hours and detected 94% of smear-positive and 50% of smear-negative cases. The single missed smear-positive case contained 1+ AFB on only 1 of 3 sputum samples analyzed by microscopy. One Xpert on an unconcentrated sputum (assuming comparable sensitivity to a concentrated sputum) would result in a median AII time of just 6.2 hours, with cost savings of up to $11466 per patient relative to AFB smear microscopy.

In contrast to an earlier study that found poor sensitivity of Xpert in a low-burden setting [30], we found Xpert-based strategies to be highly sensitive and specific relative to AFB smears, extending earlier observations from high-burden settings [9, 10]. Improved Xpert performance in our study may have resulted from higher-quality sputum samples relative to the earlier study, which relied exclusively on induced specimens.

Advantages of NAAT depend on local tuberculosis prevalence. In high-burden settings, the greater sensitivity of Xpert allows rapid diagnosis of approximately three-quarters of smear-negative, culture-positive PTB cases and offers rapid testing for rifampin resistance, enabling earlier initiation of appropriate treatment and infection control measures. In low-burden settings, the ability of Xpert to distinguish smear-positive patients with NTM infections from those with PTB is a critical advantage. As many as 30% of smear-positive cases in the United States result from NTM, yielding a substantially higher PPV for Xpert relative to smear [29]. Detecting more true negatives in a low-burden setting can avoid unnecessary empiric treatment and AII [19]. The present study supports analyses suggesting that Xpert implementation in the United States is cost-effective and can reduce AII duration [5, 31]. However, although 2 Xperts are incrementally more sensitive than 1 Xpert, a requirement for 2 sputum samples would limit the benefits on turnaround time and cost.

A single-Xpert strategy was cost-saving in a variety of sensitivity analyses, suggesting that replacement of 3 AFB smears with Xpert to determine the need for AII would result in cost savings for most US hospitals. At HMC, assuming that 200 patients are admitted with presumptive tuberculosis annually, a single unconcentrated Xpert testing strategy for AII could eliminate up to 10304 hours of AII and save as many as $2289226 for HMC annually. Assuming that 100 smears are performed for every tuberculosis case detected in the United States [2, 32], and approximately 20% of presumptive tuberculosis cases are evaluated as inpatients [5], an Xpert-based testing strategy could save the national healthcare system up to 3235799 hours of AII and $718893272 annually, if the results from this study are extrapolated to other hospitals.

This CEA considered the risk of missed tuberculosis cases by including a penalty for potential inpatient transmission [33]. Despite that consideration, both the base case and sensitivity analyses revealed that a single-Xpert strategy was cost-saving compared with 2 Xperts. A 2-Xpert strategy was equivalent to 1 Xpert only if the willingness-to-pay was close to $1750000 or tuberculosis prevalence was as high as 0.63. Nevertheless, the use of 2 Xperts is not precluded if PTB is strongly suspected or the initial specimen is suboptimal.

One limitation of this study is the hypothetical nature of the Xpert scenarios. The calculated impact of Xpert on AII duration was based on proxy measures because the test was not FDA-cleared to remove patients from AII at the time of the study. Accordingly, we have conservatively estimated the potential impact of Xpert in reducing AII, although the laboratory processing time for Xpert is in fact shorter than that for AFB smears [31, 34]. The cost-savings from a 1-Xpert strategy on unconcentrated sputum may actually be underestimated rather than overestimated. Another limitation is the lack of drug-resistant tuberculosis cases, as no resistant isolates were detected during the study period. In high-burden settings, Xpert can dramatically reduce the time to diagnosis of drug-resistant PTB [9, 10], although a rifampin-resistant result has a low PPV if the prevalence of drug-resistant tuberculosis is low [10]. A third limitation is the collection of base-case input data from a single institution and performance of the CEA from an institutional perspective, which may limit the generalizability of the findings. However, the probabilistic sensitivity analyses showed that a 1-Xpert strategy reduces costs across input ranges likely to encompass other US institutions. Given the institutional perspective, we did not consider other real-world benefits of Xpert outside the institution, such as reduced transmission, adverse events, or diminished patient satisfaction or mental health as consequences of AII [25, 35, 36]. The cost savings and benefit from a 1- or 2-Xpert strategy may be even greater from a societal perspective [37].

In resource-limited countries with high tuberculosis burdens, the WHO recommends early testing with NAAT and drug susceptibility testing, and has supported the rollout of Xpert in >110 countries [11]. However the United States has not yet implemented the routine use of NAAT for PTB diagnosis and AII discontinuation. The major barrier to adoption of NAAT in developed countries has been uncertainty regarding relevant test performance, clinical utility, and cost-effectiveness [5, 7, 20, 30, 38]. In February 2015, the FDA cleared the Xpert assay with an additional indication to enable clinicians to use 1 or 2 negative test results to discontinue AII [39]. The present study addresses these issues and provides robust support for replacing AFB smears with a single Xpert assay as the optimal strategy to guide AII in patients with presumptive PTB.

## Supplementary Material

Supplementary Data

## Notes

***Author contributions.*** F. C. F., D. R. P., and D. H. P. designed the study, with E. L. and C. W. contributing to the final protocol. F. C. F. and E. L. developed the analytical approach. A. S. C., J. F. C., E. K., and C. K. W. were responsible for data collection. A. S. C., J. F. C., C. K. W., and F. C. F. were responsible for the analysis of test performance. E. L. and C. S. were responsible for the analyses of study population, respiratory isolation duration, and cost-effectiveness. E. L., A. S. C., and J. F. C. drafted the manuscript, and F. C. F. was responsible for the final version of the paper. All authors read and authorized the final version.

***Financial support.*** This work was supported by Cepheid, which provided the GeneXpert MTB/RIF cartridges used in this study.

***Potential conflicts of interest.*** J. F. C., A. S. C., and C. K. W. have received nonfinancial support and other from Cepheid. E. L. has received personal fees, nonfinancial support, and other from Cepheid. C. S. was employed by Cepheid during the conduct of the study and has been employed by Intuitive Surgical. D. H. P. has received salary and equity derived from Cepheid, which produces the Xpert MTB/RIF test. F. C. F. has received grants, nonfinancial support, personal fees, and other from Cepheid. All other authors report no potential conflicts. All authors have submitted the ICMJE Form for Disclosure of Potential Conflicts of Interest. Conflicts that the editors consider relevant to the content of the manuscript have been disclosed.
