# Supplementary material for: Clinical Impact and Cost-effectiveness of Xpert MTB/RIF Testing in Hospitalized Patients With Presumptive Pulmonary Tuberculosis in the United States
Source: Clin Infect Dis. 2016 Dec 10;64(4):482–9. doi: 10.1093/cid/ciw803 (PMC5399932; doi:10.1093/cid/ciw803)
Supplement: Supplementary Data [file ciw803_Supplementary_Data.zip › HMC_Xpert_Appendix_20161026_clean.pdf]

APPENDIX:

Appendix Table 1. Summary of AII Duration Calculations for Testing Strategies

Testing Strategies	Test Results	Part 1<br>Data Inputs	Mean estimates (hours)	Part 2<br>Data Inputs	Mean estimates	Mean AII duration (hours)
3 Smears	Positive	Clinical data		N/A		189.18
	Negative	Clinical data		N/A		67.30
2 Smears	Positive	Clinical data		N/A		185.79
	Negative	From AII initiated to 2nd sample collection	15.31	the average time from 3rd sample collection to discharge from AII for all test negatives	32.47 hours for Possible PTB only admitting diagnosis; 42.76 hours for Additional admission diagnosis	56.57
2 Xperts	Positive	Clinical data		N/A		151.78
	Negative	From AII initiated to 2nd sample collection	15.43	the average time from 3rd sample collection time to discharge from AII	32.47 hours for Possible PTB only admitting diagnosis; 42.76 hours for Additional admission diagnosis	56.46
1 Xpert conc	Positive	Clinical data		N/A		177.32
	Negative	From AII initiated to 1st sample collection	6.95	the average time from 3rd sample collection time to discharge from AII	32.47 hours for Possible PTB only admitting diagnosis; 42.76 hours for Additional admission diagnosis	46.51
1 Xpert unconc	Positive	Clinical data		N/A		177.32
	Negative	From AII initiated to 1st sample collection	6.95	2 hours	2 hours	8.59

Abbreviations: AII, Airborne Infection Isolation; Conc, concentrated; Unconc, unconcentrated; Smears, Sputum Smear Microscopy

Footnotes:

Part 1: The time from AII initiation in hours from to the 1st, 2nd, or 3rd sample collection, depending on the evaluated strategy

Part 2: The time from the 3rd sample collection to AII discontinuation in hours

Mean AII duration calculation: sum of Part 1 and Part 2 - for example, the mean AII duration for patients with a negative test for MTB by the 1 Xpert Conc strategy -was calculated as follows: the time duration from AII initiation to the 1st sample collection plus the time duration from the 3rd sample collection to AII discontinuation.

Appendix Table 2. Clinical and Costs Inputs of Cost Effectiveness Analyses

Variables			Base case	Lower Bound	Upper Bound	Distribution	Resource
**Laboratory Cost, $**							
Sputum Smear Microscopy			6.79	6.05	7.79	Triangular	
Cost of consumables, $			0.92				1
Cost of equipment, $			1.50				1
Labor cost, $			4.00	3.26	5.00		
Cost per hours(salary and benefit)			32.03	26.09	39.97		2
Minutes per test			7.50				3
Overhead cost, $			0.37				1
Xpert MTB/RIF test cost			116.00	109.42	345.55	Triangular	
Cost of consumables, $			70.00				4
Maintenance, $			2.19	1.50	30.75		4
Cost of equipment, $			20.34	17.92	216.70		4
Labor cost, $			18.68	15.22	23.32		
Cost per hours(salary and benefit)			32.03	26.09	39.97		2
Minutes per test			35.00	35.00	35.00		3
Overhead cost, $			4.78				1
Other diagnoses, $			235.56	127.29	322.60	Triangular	
X-ray			200	110	270		
MTB culture			35.56	17.29	52.60		1
**Penalty for false negative results, $**			1213.05	1144.70	1281.40	Triangular	7
**Anti-TB treatment cost, $/per day**			50.21	17.05	296.67		3
**Non-AII Hospitalization cost rate, $/per day**			3154	500	5000	Triangular	6
**Marginal cost rate for AII , $/per day**			1527	1000	2000	Triangular	3
**AII Duration by Five Strategies, hours**†							
3 Smears	Positive	Possible PTB only admitting diagnosis	128.4	113.04	158.16	Lognormal	Clinical data*
		Additional admission diagnosis	197.76	25.2	811.92	Lognormal	Clinical data*
	Negative	Possible PTB only admitting diagnosis	57.12	20.4	113.76	Lognormal	Clinical data*
		Additional admission diagnosis	68.4	0.72	406.08	Lognormal	Clinical data*
2 Smears	Positive	Possible PTB only admitting diagnosis	128.4	113.04	158.16	Lognormal	Clinical data*
		Additional admission diagnosis	195.84	0	811.92	Lognormal	Clinical data*
	Negative	Possible PTB only admitting diagnosis	46.32	32.64	74.88	Lognormal	Clinical data*
		Additional admission diagnosis	57.84	0	106.08	Lognormal	Clinical data*
1 Xpert concentrated	Positive	Possible PTB only admitting diagnosis	120.72	97.68	158.16	Lognormal	Clinical data*
		Additional admission diagnosis	194.64	25.2	811.92	Lognormal	Clinical data*
	Negative	Possible PTB only admitting diagnosis	36	23.04	74.64	Lognormal	Clinical data*
		Additional admission diagnosis	47.76	0	323.76	Lognormal	Clinical data*
1 Xpert unconcentrated	Positive	Possible PTB only admitting diagnosis	120.72	97.68	158.16	Lognormal	Clinical data*
		Additional admission diagnosis	194.64	25.2	811.92	Lognormal	Clinical data*
	Negative	Possible PTB only admitting diagnosis	6	0	44.16	Lognormal	Clinical data*
		Additional admission diagnosis	8.88	0	282.96	Lognormal	Clinical data*
2 Xperts	Positive	Possible PTB only admitting diagnosis	104.64	40.56	158.16	Triangular	Clinical data*
		Additional admission diagnosis	168.48	25.2	811.92	Lognormal	Clinical data*
	Negative	Possible PTB only admitting diagnosis	45.6	32.64	74.88	Lognormal	Clinical data*
		Additional admission diagnosis	57.6	0	106.08	Lognormal	Clinical data*
**Extra hospitalization Duration by TB infection status, hours**							

TB infected					
Admitted for TB only	5.76	0	9.84	Lognormal	Clinical data*
Admitted for others	24.96	0	218.22	Lognormal	Clinical data*
TB not infected					
Admitted for TB only	35.52	0	481.2	Lognormal	Clinical data*
Admitted for others	138.72	0	2495.28	Lognormal	Clinical data*
**Prevalence of TB**	0.06	0.00	0.10	Triangular	Clinical data*
**Testing performance‡**					
Sensitivity of 3 Smears	0.80	0.56	0.93	Beta	Clinical data*
Specificity of 3 Smears	0.97	0.94	0.99	Beta	Clinical data*
Sensitivity of 2 Smears	0.70	0.46	0.88	Beta	Clinical data*
Specificity of 2 Smears	0.98	0.96	0.99	Beta	Clinical data*
Sensitivity of 1 Xpert	0.85	0.61	0.96	Beta	Clinical data*
Specificity of 1 Xpert	1.00	0.98	1.00	Beta	Clinical data*
Sensitivity of 2 Xperts	0.95	0.73	1.00	Beta	Clinical data*
Specificity of 2 Xperts	1.00	0.98	1.00	Beta	Clinical data*
PPV of 2 Smears	0.70	0.46	0.88	Beta	Clinical data*
NPV of 2 Smears	0.98	0.96	0.99	Beta	Clinical data*
PPV of 1 Xpert	1.00	0.77	1.00	Beta	Clinical data*
NPV of 1 Xpert	0.99	0.97	1.00	Beta	Clinical data*
PPV of 2 Xperts	0.95	0.73	1.00	Beta	Clinical data*
NPV of 2 Xperts	1.00	0.98	1.00	Beta	Clinical data*
**The proportion of admissions admitted only for TB rule-out**					
Among TB infected patients	0.25	0.09	0.49	Beta	Clinical data*
Among TB un-infected patients	0.09	0.06	0.13	Beta	Clinical data*
**The proportion of receiving anti-TB treatment**					
Among three smear positive	0.76	0.59	0.93	Beta	Clinical data*
Among three smear negative	0.05	0.03	0.08	Beta	Clinical data*

Abbreviations: MTB, *Mycobacterium tuberculosis*; TB, Tuberculosis; Smears, Sputum Smear Microscopy; Xpert, Xpert MTB/RIF; Admitted for TB only, Admitted only for evaluation of possible pulmonary tuberculosis infection; PPV, Positive Predictive Value; NPV, Negative Predictive Value.

*Data from this study, which includes HMC laboratory data and patient medical record extraction.

†For 3 smears strategy, AII duration is based on clinical data. For the other strategies, AII duration is hypothetical and estimated as follows: for Xpert MTB/RIF test positive subjects, AII duration is generated on clinical data; For Xpert MTB/RIF test negative subjects, AII duration for 2 Xperts and 2 smears is calculated via 2nd sample collection time plus the average time from 3rd sample collected to discharging from AII in patients with 3 negative smears, AII duration for 1 Xpert concentrated is calculated via 1st sample collection time plus the average time from 3rd sample collected to discharging form AII in 3 smear negative patients, and AII duration for 1 Xpert unconcentrated is calculated via 1st sample collection time plus 2 hours (approximating running the sample stat from the Emergency Room or the floor). These estimates are summarized in the Appendix Table 1.

‡Test performance of 1 Xpert unconcentrated and 1 Xpert concentrated is assumed to be the same in this analysis. The performance reported here is from 1 Xpert concentrated.

1. H.W. Choi, et al. Cost-effectiveness of Xpert MTB/RIF for diagnosing pulmonary tuberculosis in the United States. Int J Tuberc Lung Dis. 2013 17(10):1328-1335
2. http://data.spokesman.com/salaries/state/2014/all-employees/?q=laboratory+
3.Millman, Alexander J., et al. Rapid molecular testing for TB to guide respiratory isolation in the US: a cost-benefit analysis. PloS one 8.11 (2013): e79669.
4. Cepheid Schedule of Fees (2015)
5. Procedure price and savings information at Seattle hospital and cost averages. Available at http://www.newchoicehealth.com/places/washington/seattle
6.Hospital adjusted expenses per inpatient day. Available at http://kff.org/other/state-indicator/expenses-per-inpatient-day/
7. Healthcare Cost and Utilization Project (HCUP) Available at https://www.hcup-us.ahrq.gov/faststats/landing.jsp

Appendix:
The Methods of Cost Effectiveness Analysis

The sensitivity and specificity for the five testing strategies are summarized in table 2 of the manuscript. Physicians prescribed anti-TB treatment based on the results of the 3 smears, other test results and their clinical judgement. The NPV and PPV for 2 smears, 1 Xpert were used to estimate the possibility of being prescribed treatment.

AII durations were estimated by test results and whether PTB was the primary admitting diagnosis. For 3 smears and 2 Xpert assay strategies, AII durations were measured from clinical data. The details for the calculation of 2 smears, 1 Xpert with concentrated sputum and 1 Xpert with unconcentrated sputum are listed in the appendix Table 1. Additional hospital duration after AII were collected by study and calculated by TB infection and whether possible TB was the only admitting diagnosis.

The cost data used for this analysis are listed in Appendix Table 2, and include the laboratory cost, AII cost, additional non-AII hospitalization cost, treatment and penalty if applicable. We estimated lab cost of sputum smear microscopy and Xpert MTB/RIF test from the literature and updated the labor cost according to Seattle laboratory technician salaries at Harborview Hospital. A 10-year lifecycle was assumed for the Xpert platform, along with a 3% annual depreciation. We applied a cost penalty of $1213.05 to false-negative Xpert results to account for additional potential TB transmission and TB screening of exposed patients and staff associated with an earlier and/or incorrect discharge from respiratory isolation. All five strategies applied the penalty in the same manner. The decision tree for this cost effectiveness analysis is summarized in Appendix Figure 2.

Sensitivity analysis

To test the robustness of model and identify the most influential model parameters, one-way, two-way and probabilistic sensitivity analyses were performed using the ranges in the Appendix Table 2. The tornado diagrams (Appendix Figure 4 and 5) were generated to identify the most influential model

parameters. Then probabilistic uncertainty analyses were performed using Monte Carlo simulation with 10,000 simulations. The acceptance curves and ICER scatterplots (Appendix Figure 2 and 6) were reported for the results of probabilistic uncertainty analyses. Acceptance curves show the acceptance for strategies under the willingness to pay ranging from 0 to 100,000. 1 Xpert unconcentrated and 1 Xpert concentrated strategies were compared to the other strategies.

Appendix Figure 1. The Decision Tree of the Cost Effectiveness Analysis

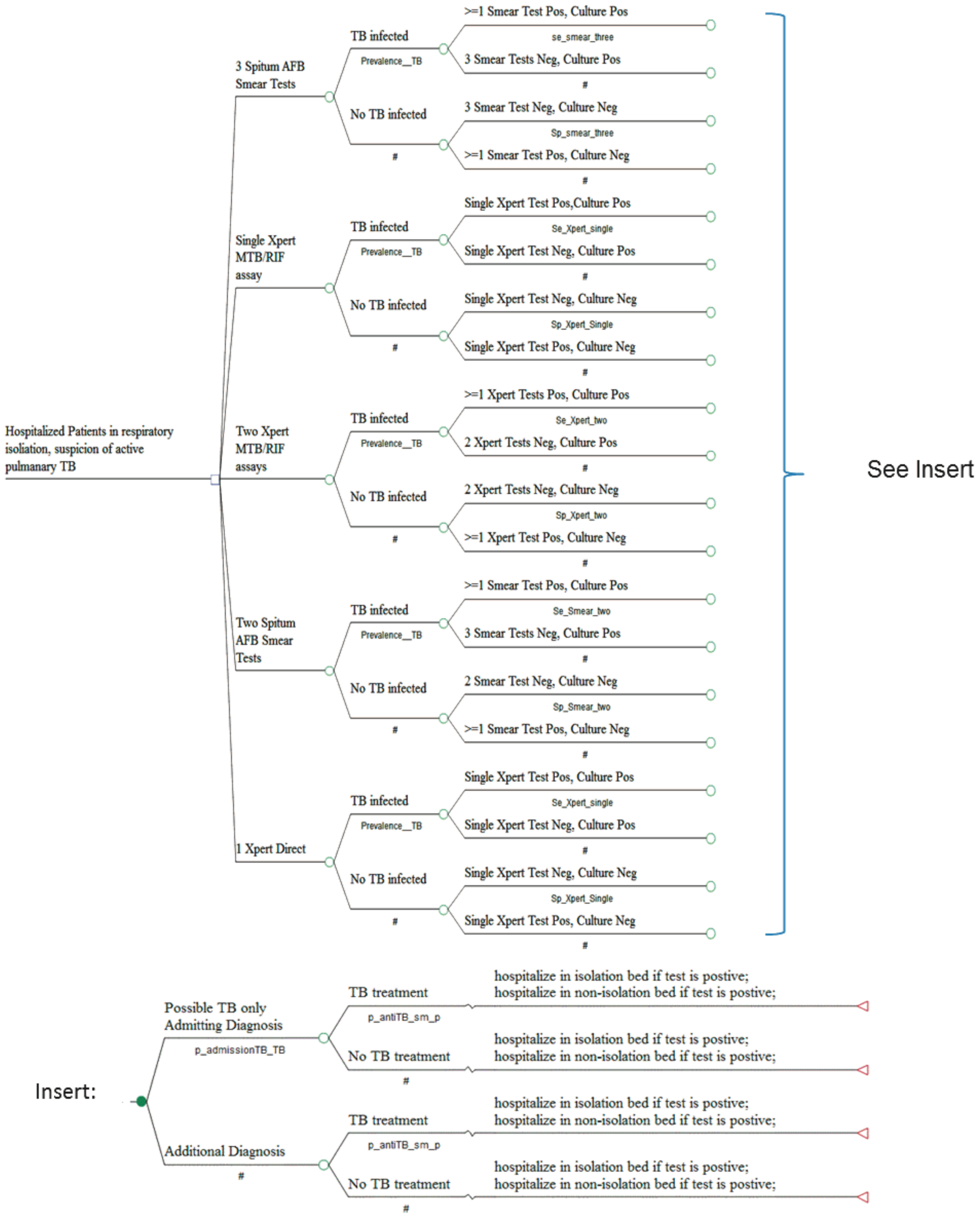

Appendix Figure 2a. The Cost Effectiveness Acceptability Curves of 4 Testing Strategies Including Xpert Unconcentrated to Determine the Need for AII

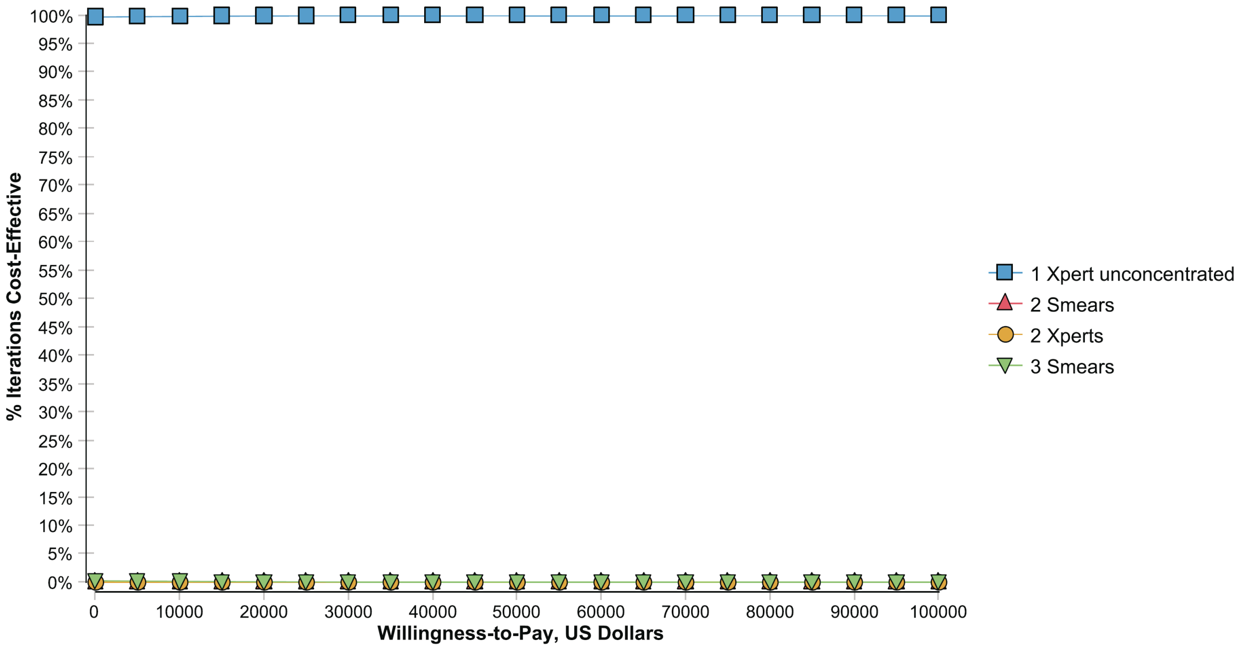

Abbreviations: AII, Airborne Infection Isolation; Xpert, Xpert MTB/RIF; Smears, Sputum Smear Microscopy; WTP, Willingness-to-Pay.

Footnote: At WTP of $50,000 USD, there is a 99.98% chance that 1 Xpert unconcentrated to be preferred.

Appendix Figure 2b. The Cost Effectiveness Acceptability Curves of 4 Testing Strategies Including 1 Xpert Concentrated to Determine the Need for AII

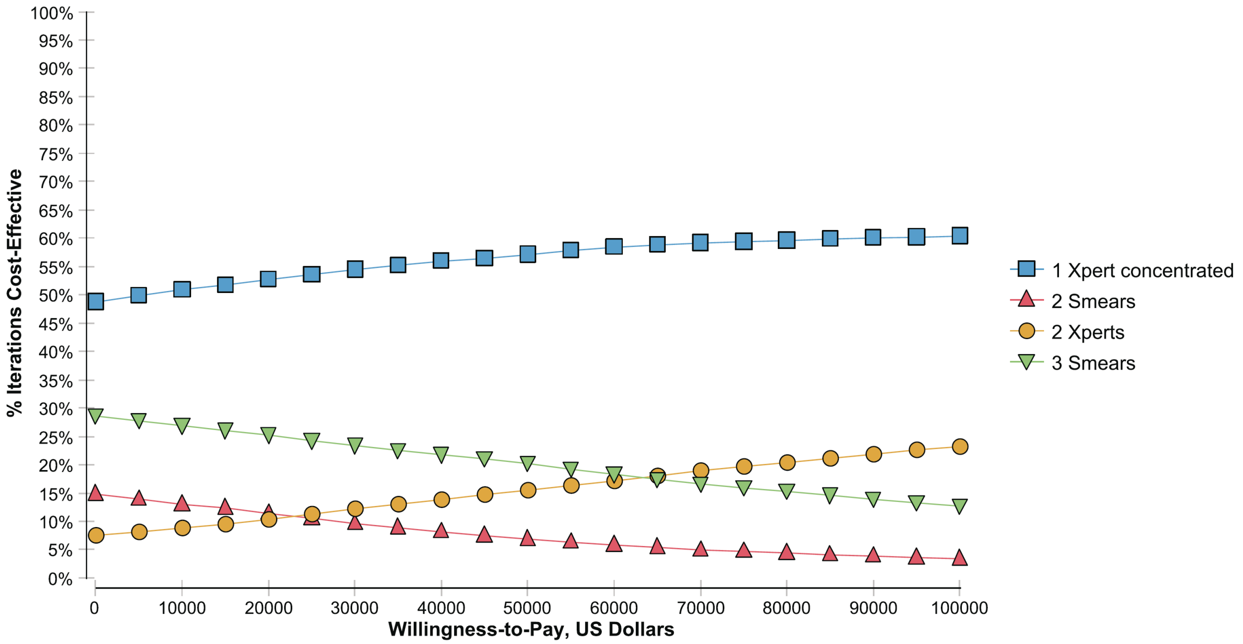

Abbreviations: AII, Airborne Infection Isolation; Xpert, Xpert MTB/RIF; Smears, Sputum Smear Microscopy; WTP, Willingness-to-Pay.

Footnote: At WTP of $50,000 USD, there is a 57.72% chance that 1 Xpert concentrated would be preferred, and there is a 16.45% chance that 2 Xperts would be preferred.

Appendix Figure 2c. The Cost Effectiveness Acceptability Curves of All 5 Testing Strategies to Determine the Need for AII

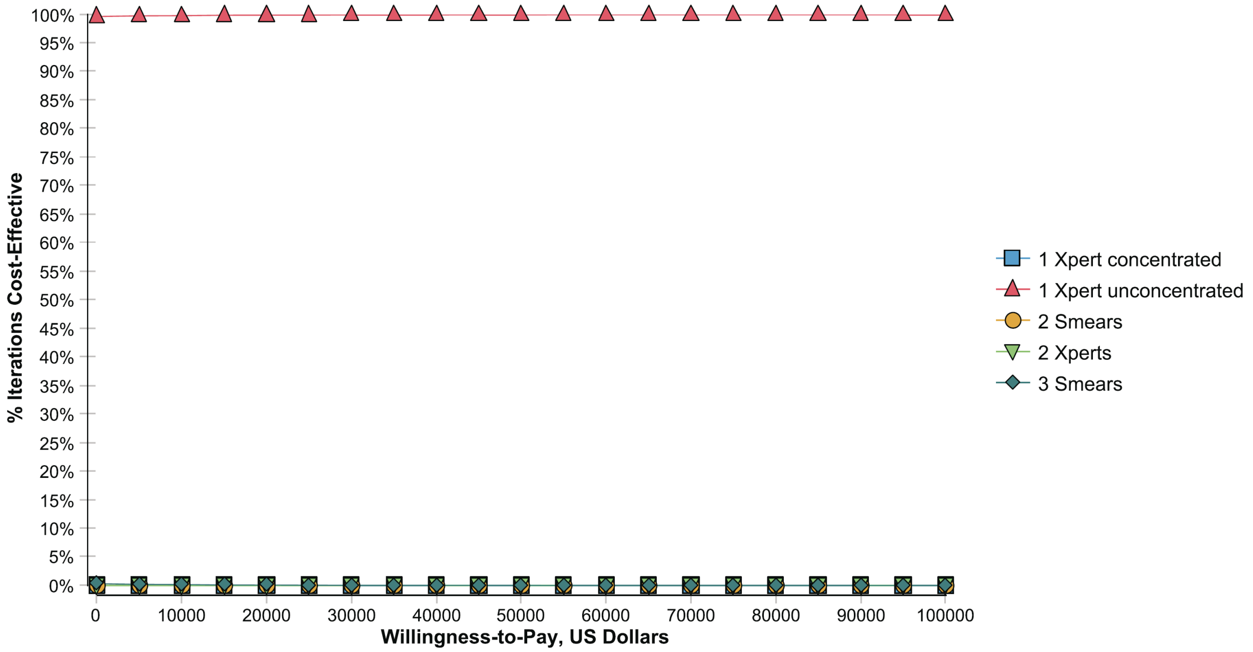

Abbreviations: AII, Airborne Infection Isolation; Xpert, Xpert MTB/RIF; Smears, Sputum Smear Microscopy; WTP, Willingness-to-Pay.

Footnote: At WTP of $50,000 USD, there is a 99.99% chance that 1 Xpert unconcentrated would be preferred.

Appendix Figure 3. The Kaplan-Meier Curves of Duration from AII Initiation to Sputum Sample Collection: including the 1st, 2nd, and 3rd Sputum Sample Collected

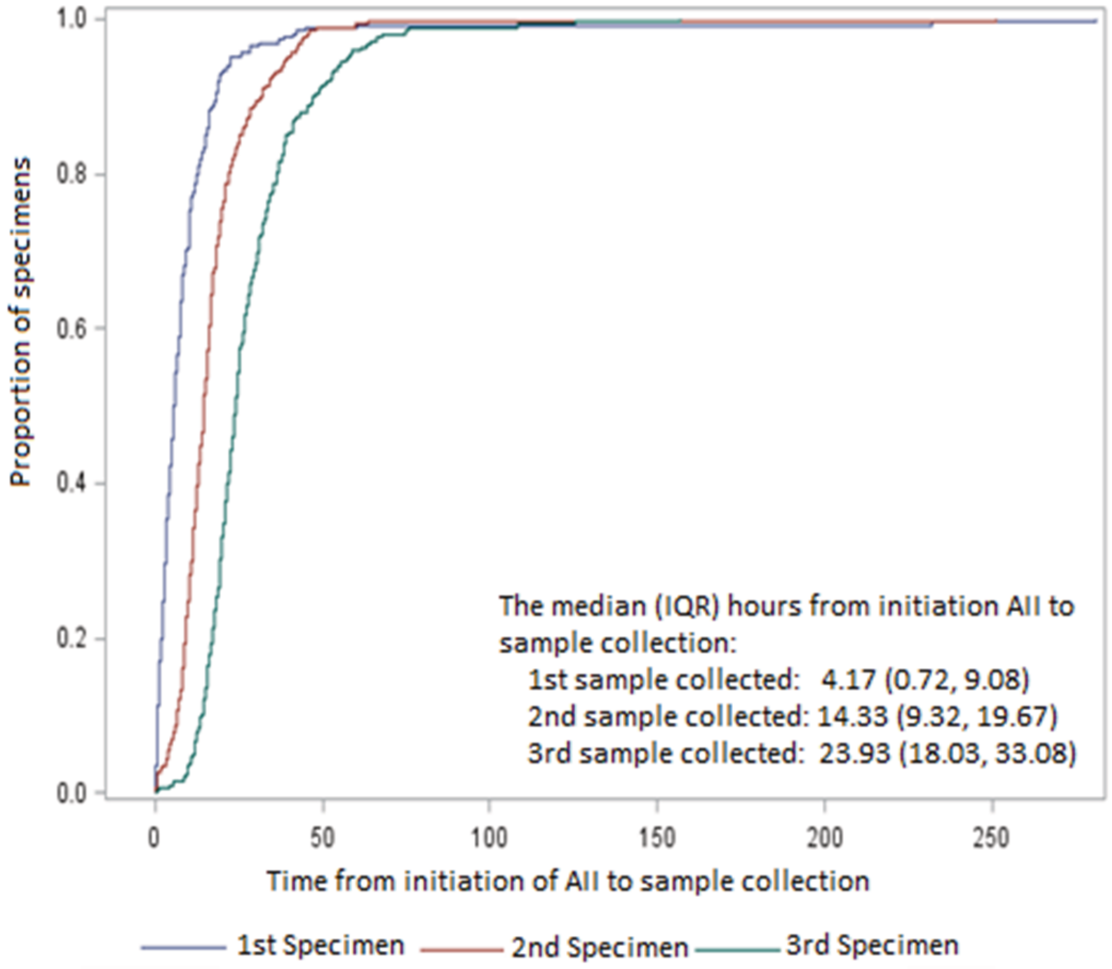

Footnote: Both Kolmogorov-Smirnov two-sample test and log-rank calculate a P-value < 0.0001.

Appendix Figure 4a. One-way Sensitivity Analysis of ICER for 1 Xpert Concentrated vs. 3 Smears

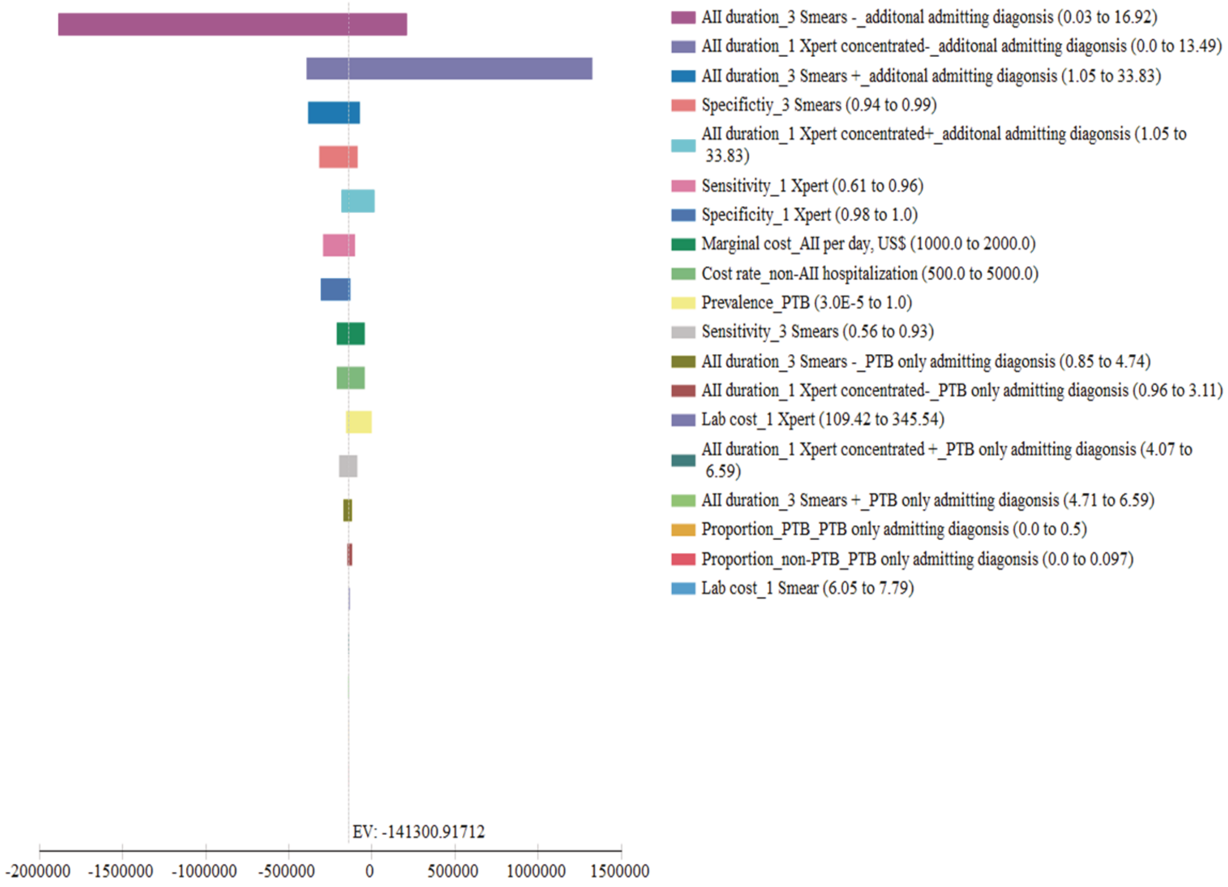

Abbreviations: AII, Airborne Infection Isolation; Xpert, Xpert MTB/RIF; PTB, Pulmonary Tuberculosis; ICER, Incremental Cost-Effectiveness Ratio; EV, Estimated Value

Appendix Figure 4b. One-way Sensitivity Analysis of ICER for 1 Xpert Concentrated vs. 2 Smears

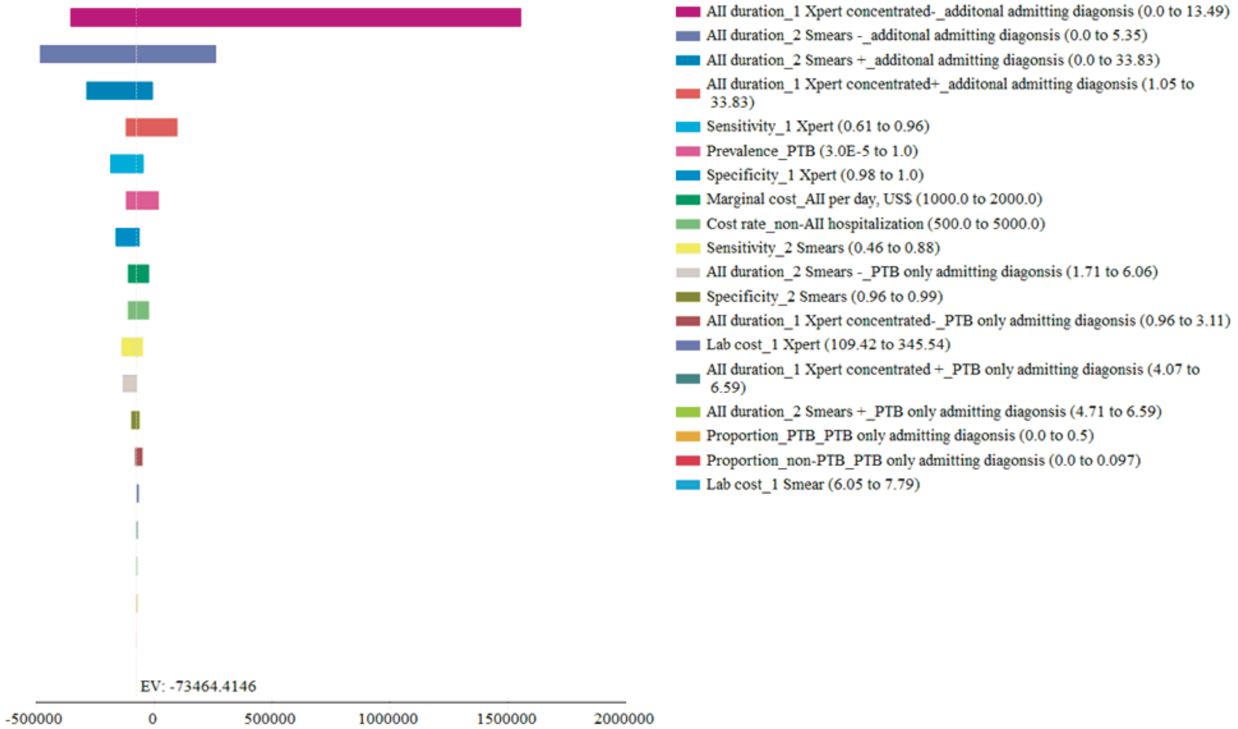

Abbreviations: AII, Airborne Infection Isolation; Xpert, Xpert MTB/RIF; PTB, Pulmonary Tuberculosis; ICER, Incremental Cost-Effectiveness Ratio; EV, Estimated Value

Appendix Figure 4c. One-way Sensitivity Analysis of ICER for 1 Xpert Concentrated vs. 2 Xperts

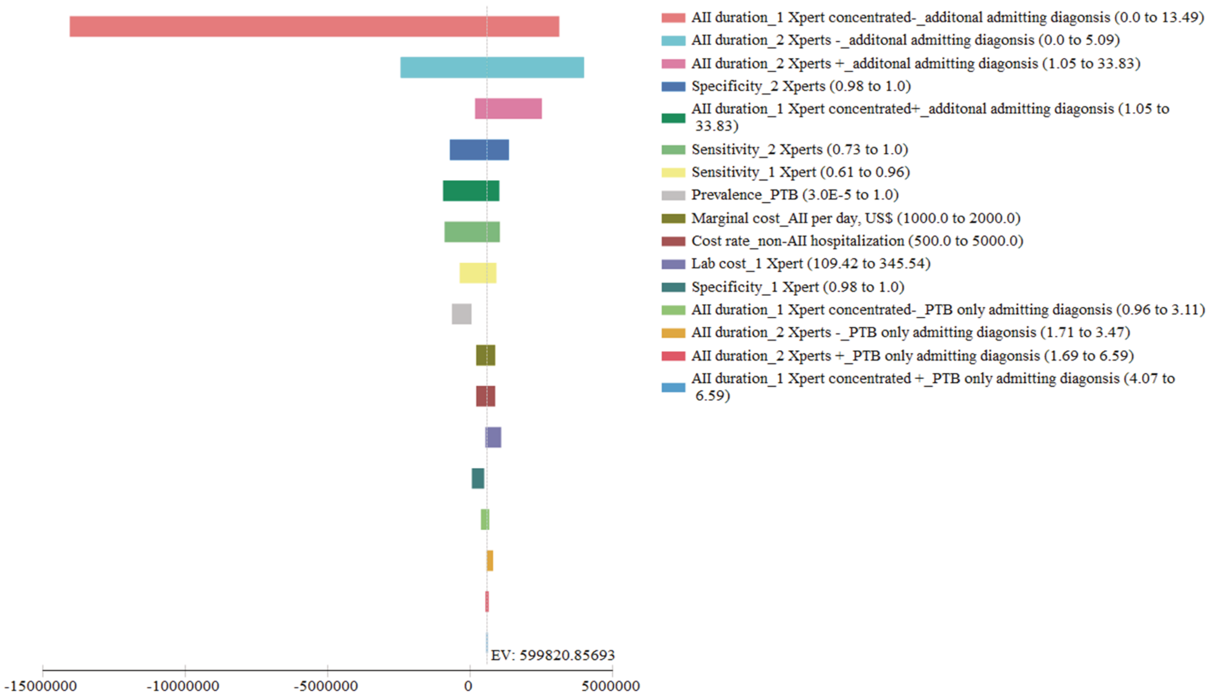

Abbreviations: AII, Airborne Infection Isolation; Xpert, Xpert MTB/RIF; PTB, Pulmonary Tuberculosis; ICER, Incremental Cost-Effectiveness Ratio; EV, Estimated Value

Appendix Figure 5a. One-way Sensitivity Analysis of ICER for 3 Smears vs. 1 Xpert Unconcentrated

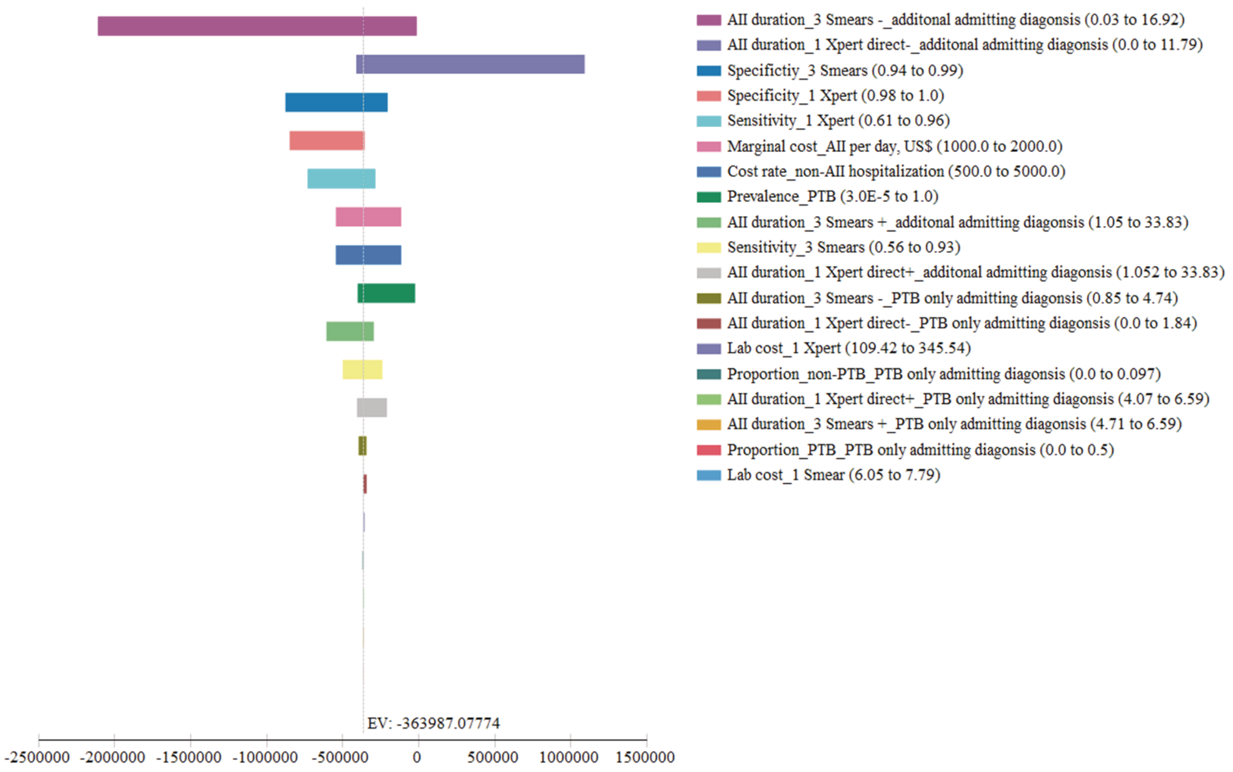

Abbreviations: AII, Airborne Infection Isolation; Xpert, Xpert MTB/RIF; PTB, Pulmonary Tuberculosis; ICER, Incremental Cost-Effectiveness Ratio; EV, Estimated Value

Appendix Figure 5b. One-way Sensitivity Analysis of ICER for 2 Smears vs. 1 Xpert Unconcentrated

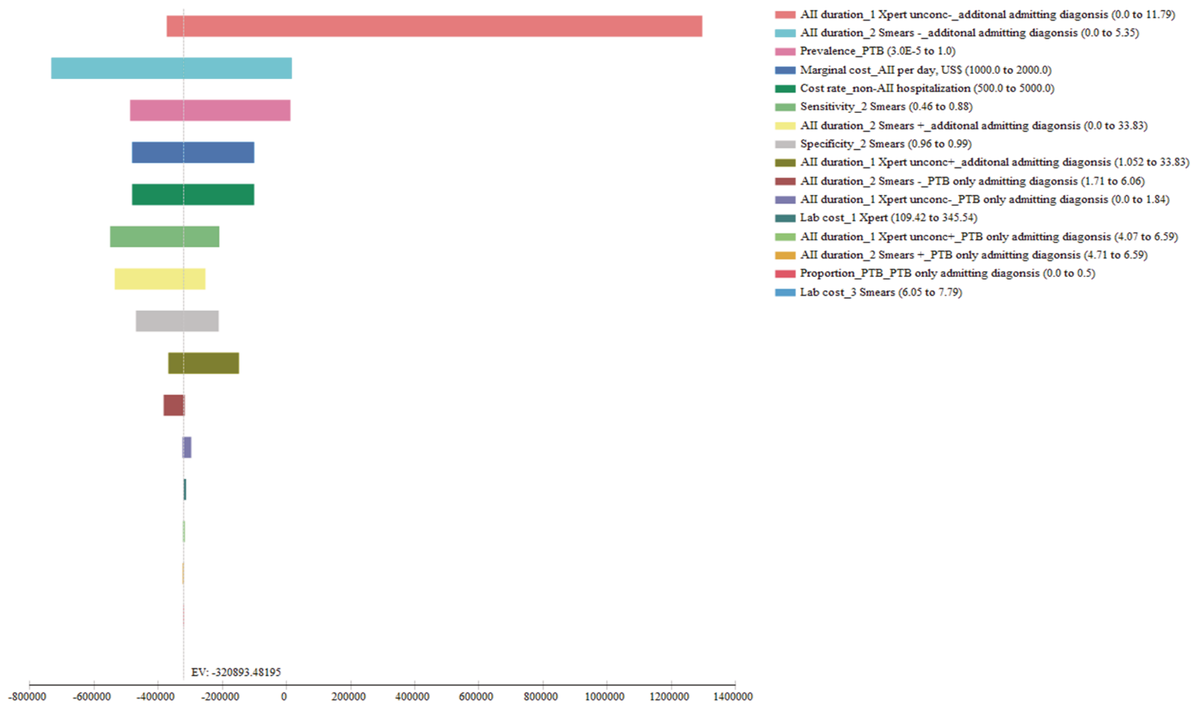

Abbreviations: AII, Airborne Infection Isolation; Xpert, Xpert MTB/RIF; PTB, Pulmonary Tuberculosis; ICER, Incremental Cost-Effectiveness Ratio; EV, Estimated Value

Appendix Figure 5c. One-way Sensitivity Analysis of ICER for 2 Xperts vs.1 Xpert Unconcentrated

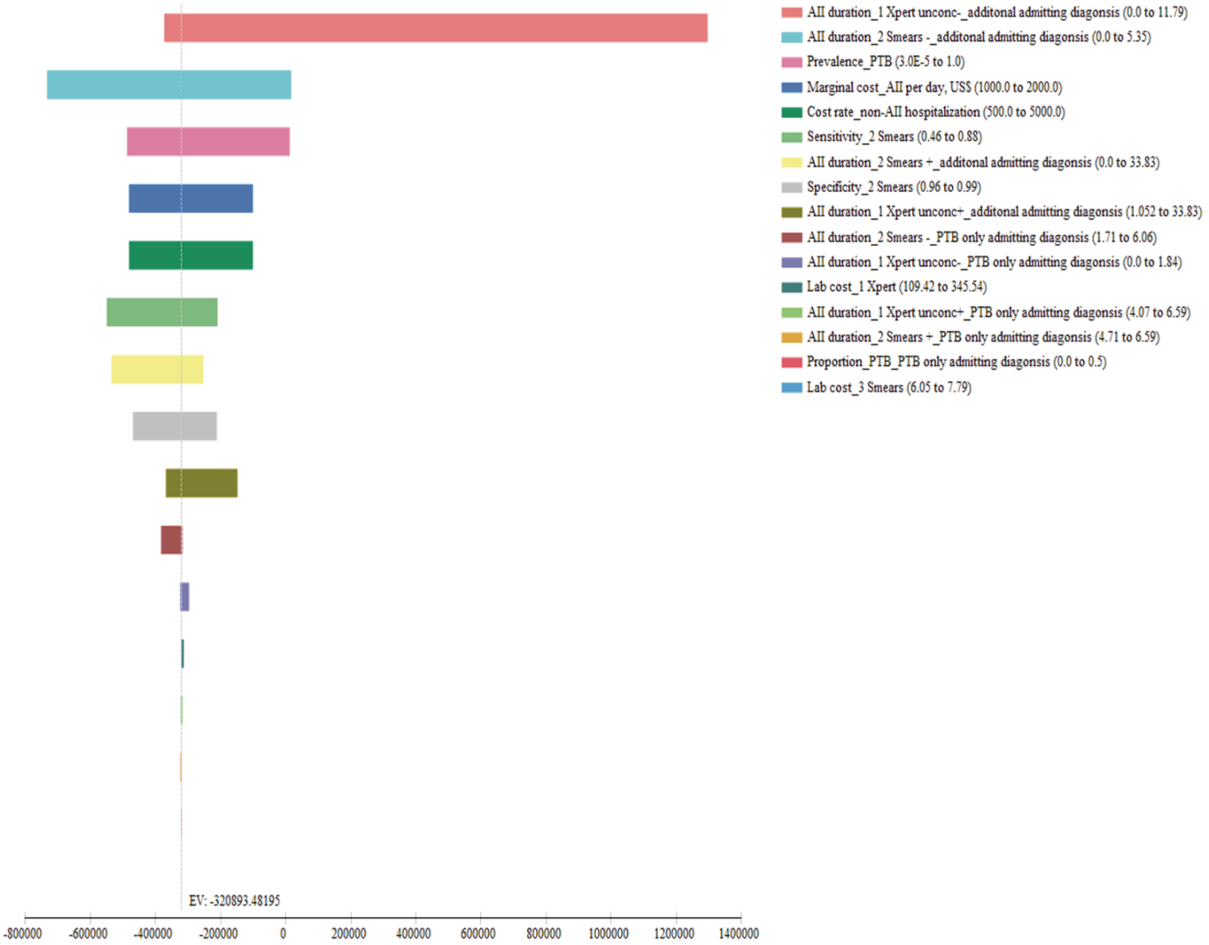

Abbreviations: AII, Airborne Infection Isolation; Xpert, Xpert MTB/RIF; PTB, Pulmonary Tuberculosis; ICER, Incremental Cost-Effectiveness Ratio; EV, Estimated Value

Appendix Figure 6a. The ICER Plane of 1 Xpert Concentrated vs. 2 Xperts from Monte Carlo Simulation*

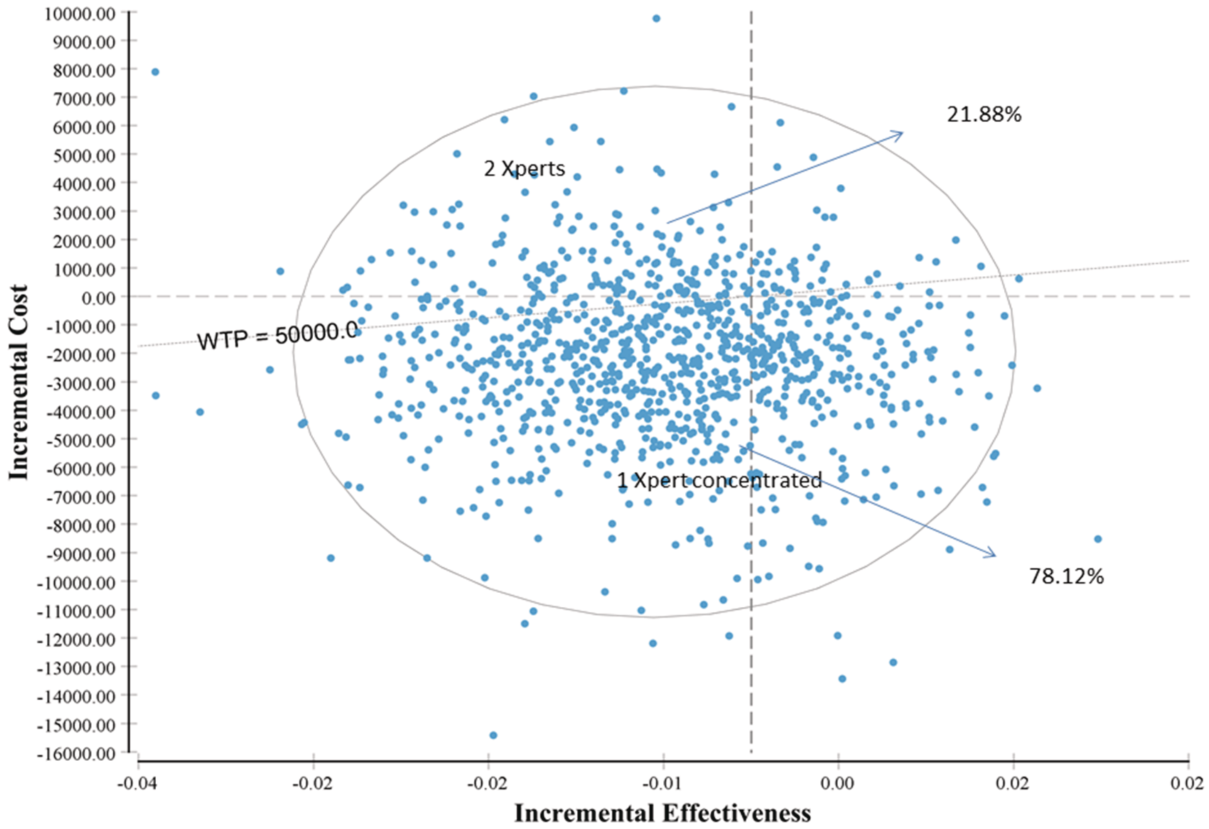

Abbreviations: ICER, Incremental Cost-Effectiveness Ratio; WTP, Willingness to Pay
*Strategy is preferred at WTP of 50,000; At WTP of 350,000, the chance of 2 Xperts preferred than 1 Xpert concentrated is 50%.

Appendix Figure 6b. The ICER Plane of 1 Xpert Unconcentrated vs. 2 Xperts from Monte Carlo Simulation*

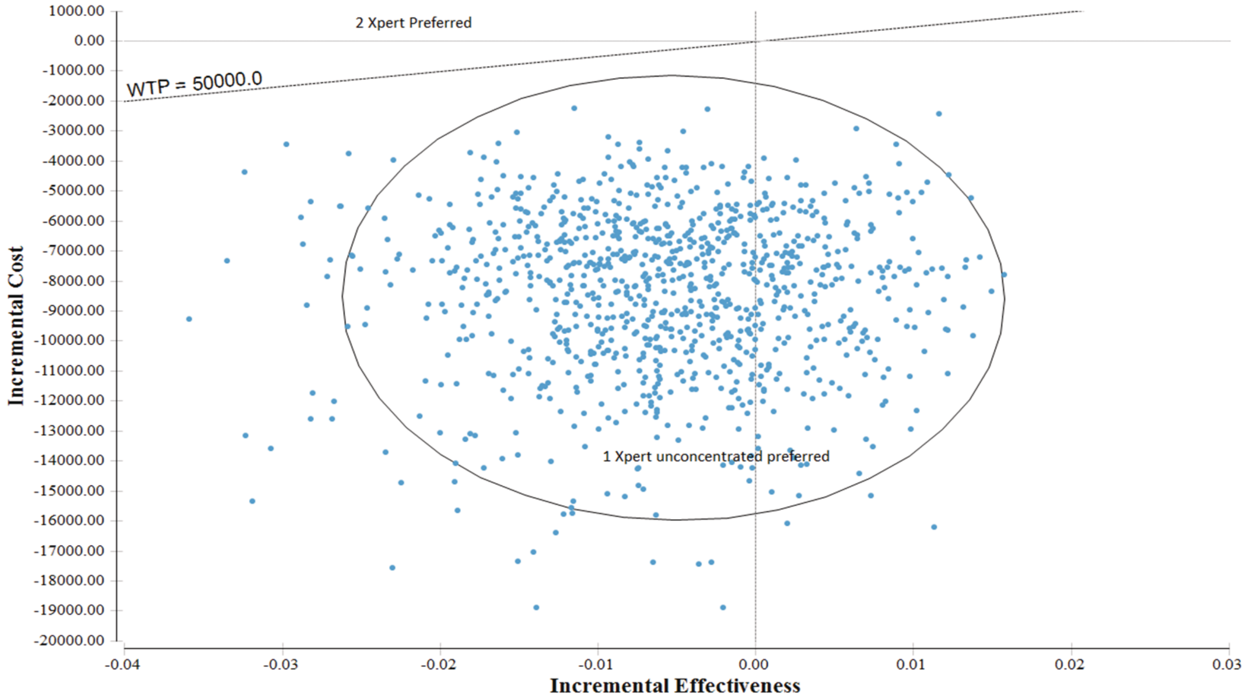

Abbreviations: ICER, Incremental Cost-Effectiveness Ratio; WTP, Willingness to Pay
*Strategy is preferred at WTP of 50,000
