# Supplementary material for: Clinical Impact and Cost-effectiveness of Xpert MTB/RIF Testing in Hospitalized Patients With Presumptive Pulmonary Tuberculosis in the United States
Source: Clin Infect Dis. 2016 Dec 10;64(4):482–9. doi: 10.1093/cid/ciw803 (PMC5399932; doi:10.1093/cid/ciw803)
Supplement: Supplementary Data [file ciw803_Supplementary_Data.zip › HMC_Xpert_Protocol_20161024_Clean.docx]

**Appendix: Study Protocol for the Laboratory Testing Procedures**

*Specimen Processing and Testing Procedures*

Specimen collection and workflow are shown in figure 1. Expectorated sputum, volume 1 – 10 mL, is collected in the presence of a nurse or physician and submitted in a sterile container.

A portion of concentrated sediment from the first sputum sample (expectorated, endotracheal suction, or induced) received for AFB culture and smear from an HMC inpatient (including ED patients who may become inpatients) will be used for analysis. This represents the volume required for analysis by either the Xpert® assay or for Standard of Care (SOC) testing. All procedures are performed within a biological safety cabinet (BSC). Per SOC, the sample (≥ 1 mL) is decontaminated and concentrated, followed by preparing smears for acid-fast staining from ~0.1 mL undiluted sediment, and resuspension of remaining sediment in a 2 mL volume, culturing 0.5 mL in liquid and 0.25 mL on solid medium, and sending 0.75 mL to the Washington State Public Health Laboratory (WSPHL) for Amplified Mycobacterium Tuberculosis Direct test, Gen-Probe (AMTD) or other NAAT testing, per current routine procedure. The leftover 0.5 mL, reserved for Xpert® testing, is relabeled with a study number, added to 1.5 mL sample treatment reagent, and tested with the Xpert® assay. Assay results are recorded on a secure log containing hospital number, patient study number and the results of AMTD assay, Xpert® assays, AFB smear, culture and susceptibility results.

HMC Infection Control personnel will ensure that second and third sputum specimens are collected for AFB culture and smear on subsequent consecutive days or at least eight hours apart. The second sputum sample will be processed as for the first sample, while the entire third specimen will be processed per SOC Testing described below.

# *Standard of Care (SOC) Testing*

1. Each sputum specimen is processed by the NALC-NaOH decontamination and concentration method (Kent & Kubica, 1985), using the AlphaTec™ (Vancouver, WA) reagent system: NAC-PAC *Red* decontamination solution, providing final concentration of 1% NaOH, NPC-67 neutralizing buffer,), centrifugation at 5000 xg for 15 minutes, and resuspension in Pellet Resuspension Buffer. Processing is performed on batched specimens once daily at 9AM. Auramine acid-fast stain results for processed specimens are reported the same day. Samples received after 9AM are refrigerated and processed the following day.
2. Two smears are prepared directly from the concentrated sputum sediment.
3. The remaining sediment is suspended in Pellet Resuspension Buffer and 0.75 mL of the sediment suspension inoculated to each of two media: 0.25 mL on one Middlebrook 7H11 agar plate and 0.5 mL into one 7 mL BBL™MGIT™ (Mycobacterium Growth Indicator Tube) broth (supplemented with 0.8 mL PANTA/OADC supplement.
4. A 0.75 mL aliquot of the resuspended sediment is refrigerated and sent to the WSPHL TBLaboratory for AMTD testing, performed on Tuesdays and Thursdays.
5. Cultures:
   1. M7H11 agar culture media is incubated at 37°C in 5-10% CO2, observed twice a week the first two weeks, then weekly for eight weeks.
   2. MGIT broth culture media is incubated at 37°C in the MGIT 960 automated incubation and growth detection instrument for six weeks.
6. Acid-fast stains: One smear per patient is stained with auramine acid-fast stain. The entire area of the smear is read at low power magnification (200X) before reporting a smear as negative. The second unstained smear is stored for possible future testing.
7. AFB positive cultures:
   1. *M. tuberculosis* (MTB) complex isolates are identified to the complex level by Gen-Probe Accuprobe, and to species by pyrazinamidase (PZA). MTB complex isolates with negative PZA results are further tested by niacin and nitrate reductase phenotypic tests to differentiate *M. bovis* and pyrazinamide resistant *M. tuberculosis.*
   2. Other mycobacteria are identified with Gen-Probe Accuprobes for *M. avium* complex and *M. gordonae*, or by DNA sequencing at the University of Washington Medical Center Clinical Microbiology Laboratory.
8. *M. tuberculosis* complex susceptibilities: The first isolate per patient is tested by the modified proportion method in 7H9 broth using the MGIT 960 system. Any resistant results are confirmed by repeat testing at HMC, and by the proportion method in 7H10 agar at the WSPHL TB Laboratory.

# *Xpert® MTB/RIF→ Specimen Handling*

1. Testing will use a 0.5 mL aliquot leftover after concentration and use for AFB smears, culture and NAAT testing by the WSPHL, from the first sputum sample (expectorated, endotracheal suction, or induced, and in ≥1 mL volume) received for AFB culture from an HMC inpatient (including emergency department patients who may become inpatients).
2. The aliquot reserved for Xpert® testing is relabeled with a study number and tested directly with the Xpert® assay soon after receipt, with the exception that samples received between 9:30PM and 6AM are tested during the next dayshift.
3. If the Xpert® assay cannot be performed immediately, specimens are stored at RT for up to 24hrs or at 2-8°C for up to 5d.
4. Mycobacteria isolated from study specimens are stored frozen at ≤70ºC for the duration of the study, and some may be frozen for an indefinite time period, per above SOC protocol.

# *Xpert® MTB/RIF→ Assay Procedure*

1. Open sputum sample tube and process sample for assay in a BSC.
2. Open Sample Treatment Reagent (SR, or elution buffer) bottle, and add 1.5 mL into sample tube. Recap sample tube and discard SR bottle. SR: specimen ratio should be 3:1.
3. Shake tube *vigorously* 20 times.
4. Incubate tube at room temperature for 10 minutes.
5. Then shake tube *vigorously* 20 times again.
6. Incubate 5 more minutes at room temperature, for a total of 15 minutes.
7. Open cartridge lid. Use sterile pipette to transfer entire volume of digested 2 mL sample into the opening of the Xpert® cartridge, and close lid.
8. Load the cartridge into the GeneXpert® instrument
9. Initiate testing by selecting the automated protocol from the included software. Test results print automatically when the test is completed, < 2 hours.
10. If the initial Xpert® result is “INVALID,” “ERROR” or “NO RESULT,” retest the specimen. An “R” will be added to the sample ID to designate a re-tested specimen.
11. Store leftover specimens at ≤75°C.

# *Xpert® MTB/RIF→ External Control Runs*

Negative and positive control samples will be provided by Cepheid. One negative and positive external control will be tested when new lots of Xpert® MTB/RIF cartridges are received. If either control is indeterminate or incorrect, the test will be repeated using a new control vial, Xpert® cartridge, and liquid reagents, in the same Xpert® module.
